# Whole-exome sequencing capture kit biases yield false negative mutation calls in TCGA cohorts

**DOI:** 10.1371/journal.pone.0204912

**Published:** 2018-10-03

**Authors:** Victor G. Wang, Hyunsoo Kim, Jeffrey H. Chuang

**Affiliations:** 1 The Jackson Laboratory for Genomic Medicine, Farmington, CT, United States of America; 2 University of Connecticut Health Center, Department of Genetics and Genome Sciences, Farmington, CT, United States of America; Ohio State University Wexner Medical Center, UNITED STATES

## Abstract

The Cancer Genome Atlas (TCGA) provides a genetic characterization of more than ten thousand tumors, enabling the discovery of novel driver mutations, molecular subtypes, and enticing drug targets across many histologies. Here we investigated why some mutations are common in particular cancer types but absent in others. As an example, we observed that the gene *CCDC168* has no mutations in the stomach adenocarcinoma (STAD) cohort despite its common presence in other tumor types. Surprisingly, we found that the lack of called mutations was due to a systematic insufficiency in the number of sequencing reads in the STAD and other cohorts, as opposed to differential driver biology. Using strict filtering criteria, we found similar behavior in four other genes across TCGA cohorts, with each gene exhibiting systematic sequencing depth issues affecting the ability to call mutations. We identified the culprit as the choice of exome capture kit, as kit choice was highly associated with the set of genes that have insufficient reads to call a mutation. Overall, we found that thousands of samples across all cohorts are subject to some capture kit problems. For example, for the 6353 samples using the Broad Institute’s Custom capture kit there are undercalling biases for at least 4833 genes. False negative mutation calls at these genes may obscure biological similarities between tumor types and other important cancer driver effects in TCGA datasets.

## Introduction

The Cancer Genome Atlas (TCGA) has been a valuable resource for shining light on tumor genetic and molecular biology, allowing for the move towards targeted therapy oncology clinical trials like NCI’s MATCH [1]. One of TCGA’s many strengths is the coverage and depth of their whole-exome sequencing (WES) protocol; the average of approximately 100x coverage [2] has been used to confidently call mutations even at allele frequencies of 0.2 or below using MuTect [3]. This mutation calling power has enabled important translational research such as identifying targetable driver mutations [4]. The scope of TCGA suggests it potential value for identifying systematic driver effects across cancer types. At the same time, this broad scope makes it more susceptible to measurement errors. For example, Buckley et al. found technical artifacts in TCGA germline samples due to whole chromosome amplification resulting in spurious indel calls [5].

To understand which processes are most important in cancer development, it is critical to have accurate assessments of which mutations recur in different cancer types. However, there may be other spurious mutation annotations in TCGA due to systematic biases. For example, *CCDC168* is a protein-coding gene with poorly understood function known to be mutated in several cancer types. Studies based on TCGA data have reported that this locus is susceptible to microsatellite-instability events resulting in frameshift mutations in colorectal cancer but not in gastric cancer, and this has been interpreted as a functional distinction between the tumor types [6]. This finding is puzzling, as a subtype of stomach adenocarcinomas is subject to microsatellite instability [7], which would provide a mechanism for *CCDC168* frameshift mutations to occur in stomach adenocarcinomas as well. We therefore hypothesized that the lack of *CCDC168* mutations in stomach adenocarcinoma might be due to measurement bias.

In this work, we have investigated whether *CCDC168* mutations and other TCGA mutations are impacted by measurement bias by considering features in each cancer sample associated with a failure to call mutations. We show that measurement bias associated with the exome capture platform explains the *CCDC168* effect. Moreover, we demonstrate how these platform biases affect mutation calling throughout TCGA data. Our results indicate that potentially false negative somatic mutation calls due to insufficient coverage recurrently impact at least 701 genes. Over 8000 samples across a wide variety of TCGA tumor cohorts used the implicated capture kits. Due to these false negatives, different tumor types may be more mutationally similar than previously reported, and the impact of these genes on cancer may have been underestimated.

## Results

### *CCDC168* shows a systematic lack of mutations in stomach adenocarcinoma

To better understand why TCGA stomach adenocarcinoma samples (STAD) lack *CCDC168* mutations, we first manually inspected read depth in individual STAD samples. This revealed low numbers of reads aligning to the *CCDC168* locus (S1 Fig). We then analyzed this behavior across all STAD samples, which showed that overall 425 of 441 STAD tumor samples had fewer than 1,000 aligned reads along the gene, with 50% of samples having 12 or fewer reads (Table A in S1 Tables). Given the exon length of 21,470 base pairs (Methods), a read count of 1000 would yield only 2.7x coverage over the gene. We calculated the average exon coverage across *CCDC168*, and this showed no samples exceeding 6.4 (Table A in S1 Tables and A in S2 Fig). Mutation callers typically use 30x coverage to call an SNV in short-read sequencing [8]. Therefore, the lack of called mutations in *CCDC168* can be attributed to insufficient coverage at the locus.

### Some genes systematically lack mutation calls across multiple cohorts

We then searched for other genes with a systematic lack of mutation calls and analyzed whether they had cohort-specific biases. To do this we analyzed non-silent mutations using all TCGA MAF files. 22017 of 22022 genes had at least one cohort where no mutation was called in any of the cohort samples. Here we use the term cohort to refer to TCGA samples from different tissues, e.g. stomach adenocarcinoma, colon adenocarcinoma, etc. To distinguish potential genes subject to cohort-specific measurement biases from those with true low mutation rates, we considered only genes that met minimum criteria for mutational prevalence in the overall TCGA set (Methods). This yielded 136 high-confidence genes having strong cohort-specific biases for mutational absence (Table B in S1 Tables). Several genes showed bias across multiple cohorts. For example, *CCDC168* lacked mutation calls in 17 cohorts, i.e. over half of all cohorts in TCGA. Other genes with similar behavior included *SETD1B* and *SOX11*, which lacked called mutations in 20 and 14 cohorts, respectively.

### Multiple genes lack sufficient coverage for mutation calling

**Fig 1:**
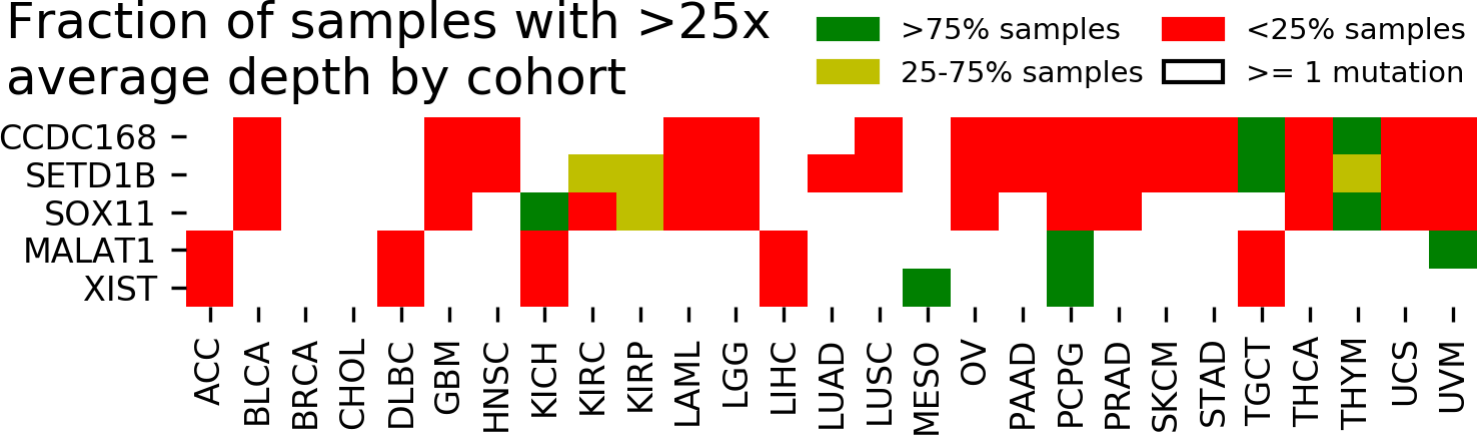
Cohorts with insufficient coverage to call mutations. Table of the five genes of interest and coverage status by cohort. Coverage status is determined by the fraction of samples in the cohort which have sufficient coverage in the gene to call a mutation. Cohorts with at least one sample with a called mutation in the gene were not considered and are labeled white.

We next explored whether these cases of mutational absence were due to a systematic lack of coverage. To test this, we downloaded reads from TCGA WES samples aligned to the 136 genes (Methods). We set a minimum average depth threshold of 25x across all bases in a gene’s canonical exons to assess if a gene had sufficient coverage to call a mutation. At this threshold, Mutect has a sensitivity approaching 0.99 for an allele fraction of 0.3 [3], which approximates the 60% tumor purity requirement for TCGA [9] for a heterozygous mutation. We saw that *CCDC168* systematically lacked sufficient coverage to call mutations in 14 other cohorts (Fig 1) at the 25x threshold. Notable exceptions were testicular germ cell cancer (TGCT) and thymoma (THYM), for which every sample in each cohort had sufficient coverage yet no *CCDC168* mutations were called, indicating these to be true negative findings.

Four other genes had similar patterns as *CCDC168*, i.e. where large numbers of samples within multiple cohorts had insufficient coverage to call mutations. We defined *undercovered cohorts* as those in which over 75% of samples had insufficient coverage to call mutations for a given gene. The four genes which had multiple undercovered cohorts were the long non-coding RNAs (lncRNAs) *MALAT1* and *XIST* and the protein-coding genes *SETD1B* and *SOX11*. The undercovered cohorts of the three protein coding genes had strong similarities, with PRAD tumors in particular uniformly showing insufficient coverage at all three gene loci (Fig 1 and B in S2 Fig). Additionally, *MALAT1* and *XIST* shared identical undercovered cohorts (Fig 1 and C in S2 Fig). Interestingly, undercovered cohorts of the three protein-coding genes and those of the lncRNAs were mutually exclusive (Fig 1), suggesting distinct reasons for these behaviors.

### Capture kit choice explains undercovered cohorts

These gene- and cohort-specific behaviors suggested that systematic sequencing quality issues might be responsible for the insufficient coverage and lack of mutation calls. A potential culprit is the exome capture kit, which we hypothesized had gene-specific inefficient pulldown in some cohorts. To investigate this, we retrieved information on each sample’s capture kit using the NIH’s Genomic Data Commons (GDC) Search and Retrieval API (Methods). We found that cohorts annotated as assayed with the Custom V2 Exome Bait capture kit exclusively were undercovered for at least one of *CCDC168*, *SETD1B*, or *SOX11* (Tables C and D in S1 Tables). The Custom V2 Exome Bait capture kit appears to be a proprietary exome capture kit manufactured by Agilent and used by the Broad Institute for TCGA (Table 1). The TGCT and THYM cohorts used different capture protocols, and neither were undercovered for these genes despite no called mutations. The common capture kit for undercovered cohorts in the two lncRNAs was the SeqCap EZ HGSC VCRome developed by Roche NimbleGen (now known only as Roche) and used by Baylor University. However, the three cohorts with sufficient coverage of the lncRNAs in all samples used other capture protocols. All samples in TCGA used paired-end sequencing chemistry, negating it as a confounder to explain the observed differences between tumor histologies. These associations provide strong evidence that capture kits have biases that lead to failure to call mutations in some cohorts.

**Table 1:** Capture kits used in TCGA.

Manufacturer	Exon Capture Kit Name	Bait Type	Probe Length	# Samples	User
Agilent	Custom V2 Exome Bait	Unknown	Unknown	6353	BI
Agilent	SureSelect Human All Exon 38 Mb v2	cRNA	120 (Adjacent)	493	WUGSC
Agilent	SureSelect Human All Exon 50 Mb	cRNA	120 (Adjacent)	7	WUGSC
Agilent	SureSelectXT Human All Exon V5	cRNA	120 (Adjacent)	83	WUGSC
Roche NimbleGen	Gapfiller_7m	Unknown	Unknown	48	BCM
Roche NimbleGen	SeqCap EZ HGSC VCRome	Unknown	Unknown	1395	BCM
Roche NimbleGen	SeqCap EZ Human Exome Library v2	DNA	60–90 (Tiled)	1094	WUGSC
Roche NimbleGen	SeqCap EZ Human Exome Library v3	DNA*	60–90 (Tiled)*	1367	WUGSC

Eight exon capture kits were used in TCGA, with the Custom V2 Exome Bait kit used by the Broad Institute accounting for a majority of WXS samples. Attributes are derived from *Sulonen et al*. [10], except for Agilent’s SureSelectXT Human All Exon V5 [11]. Adjacent probes are non-overlapping whereas tiled probes overlap in the targeted regions. Unknown indicates information not publicly-available.

Attributes with an asterisk (*) for the SeqCap EZ Human Exome Library v3 kit are not reported and assumed to be the same as the previous version.

BI = Broad Institute. WUGSC = Washington University Genome Sequencing Center. BCM = Baylor College of Medicine.

**Fig 2:**
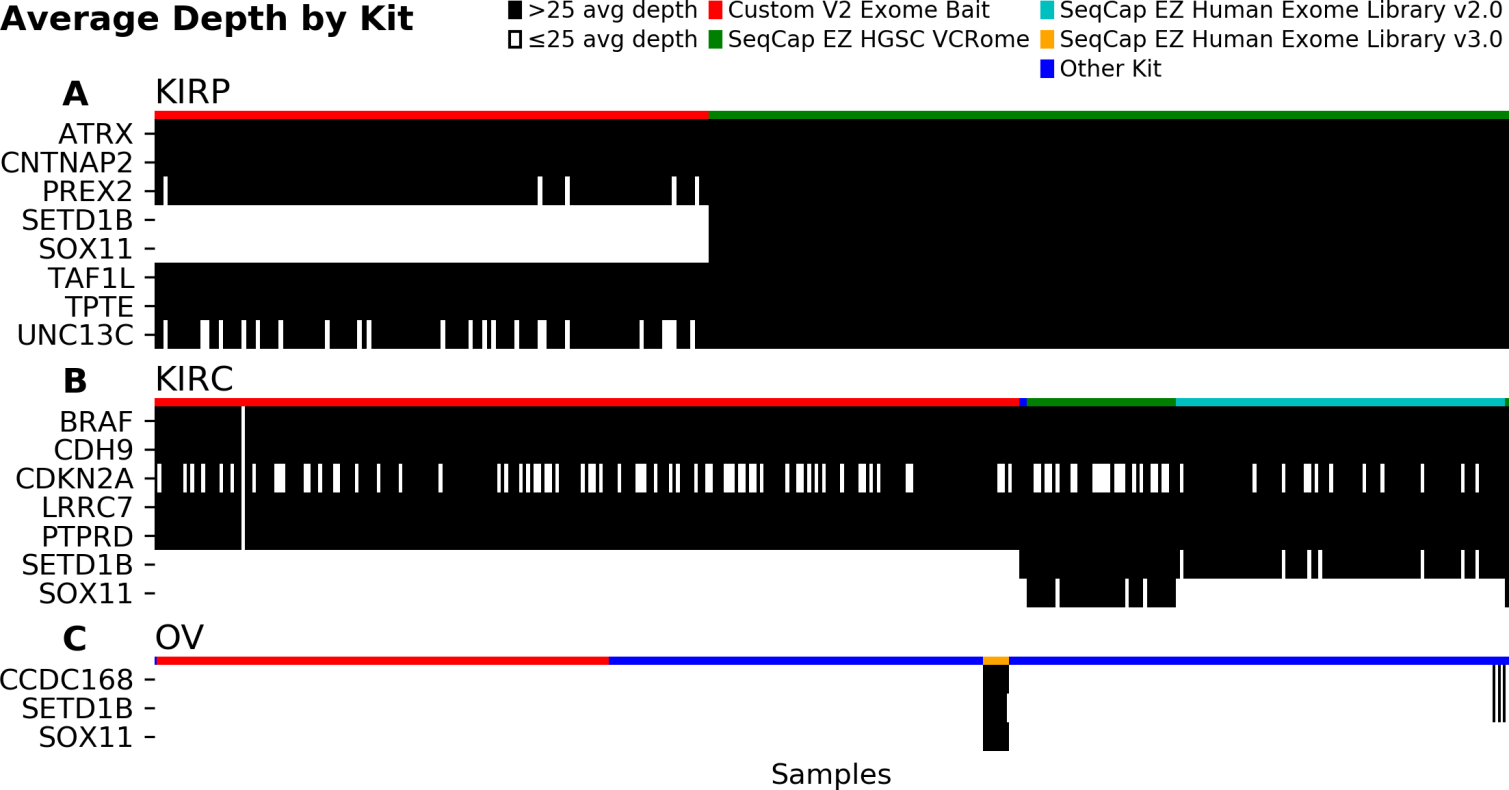
Capture kit explains insufficient coverage. In cohorts assayed by heterogeneous capture kits, samples with insufficient coverage can be differentiated by kit. In KIRP (A), samples using the Broad’s Custom V2 Exome Bait kit have insufficient coverage in *SETD1B* and *SOX11*, whereas samples that use other kits have sufficient coverage. The KIRC cohort (B) shares this behavior, with Roche NimbleGen’s SeqCap EZ Human Exome Library v2.0 kit also yielding insufficient coverage in *SOX11*. In OV (C) all the kits except Roche NimbleGen’s SeqCap EZ Human Exome Library v3.0 kit yielded insufficient coverage of the three protein-coding genes.

This kit-specific effect also explained variations in coverage within the cohorts that exhibited complete absence of mutations in at least one of the five genes. For example, in the kidney renal papillary cell carcinoma (KIRP) cohort, the usage of the custom kit explained all 120 cases with insufficient coverage at the *SETD1B* and *SOX11* loci (Fig 2A). For the kidney renal clear cell carcinoma (KIRC) cohort, an additional 90 samples had insufficient coverage for other kits (Fig 2B), notably for *SOX11* when studied with Roche NimbleGen’s SeqCap EZ Human Exome Library v2.0 kit. In ovarian serous cystadenocarcinoma (OV), where 512 samples lacked sufficient coverage in one of the three protein-coding genes, the 10 samples with sufficient coverage were all measured with Roche NimbleGen’s SeqCap EZ Human Exome Library v3.0 kit (Fig 2C). These findings show a clear association between exome capture kits and samples with insufficient coverage at the five genes of interest.

### Underestimation of gene mutation rates in cohorts

**Fig 3:**
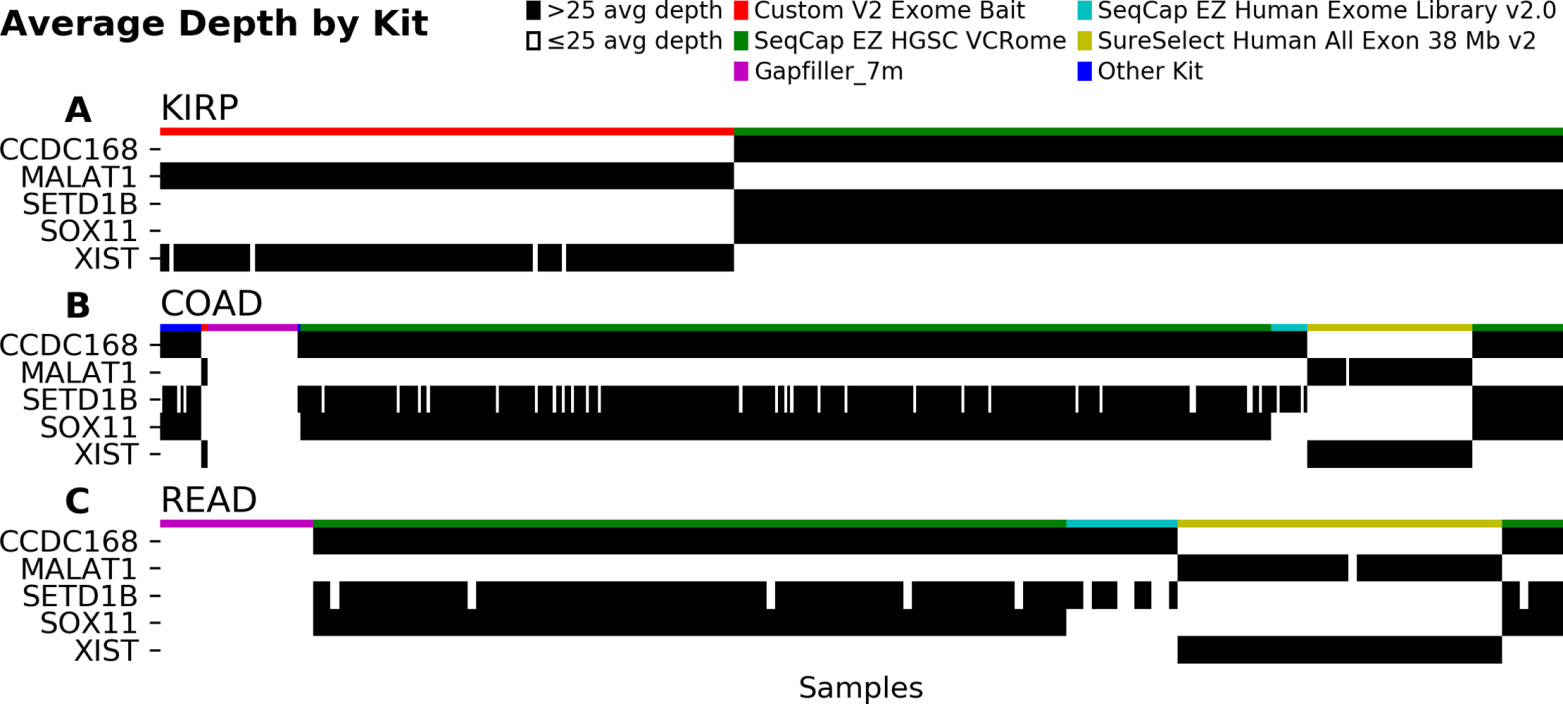
Capture kit is associated with samples lacking sufficient coverage in 5 recurrent genes. The KIRP cohort (A) uses both the Custom V2 Exome Bait kit, which is associated with insufficient coverage in the 3 protein-coding genes, and the SeqCap EZ HGSC VCRome kit, which associates with insufficient coverage in the lncRNAs. In both the COAD (B) and READ (C) cohorts, samples using the SeqCap EZ HGSC VCRome kit have insufficient coverage on the lncRNAs. Gapfiller_7m and SureSelect Human All Exon 38 Mb v2 are also associated with systematic biases in coverage on the five genes.

We then expanded our scope to include TCGA cohorts where mutations had been observed, focusing on the five genes described above. Again we found that coverage bias was impacted by capture kit choices. The KIRP cohort used the Custom V2 Exome Bait kit, SeqCap EZ HGSC VCRome kit, and derivatives such as the VCRome V2.1-PKv1 kit. As expected from the intercohort analysis, the samples using the Custom V2 Exome Bait kit had insufficient coverage at the three protein coding loci, whereas the samples using the SeqCap EZ HGSC VCRome kits had insufficient coverage at the two lncRNA loci (Fig 3A). This effect had not been clear at the cohort level as *CCDC168*, *MALAT1*, and *XIST* each had at least one mutation called in the KIRP cohort.

As other examples, we also checked the extent of the capture kit effect in the COAD (Fig 3B) and rectum adenocarcinoma (READ) (Fig 3C) cohorts. We chose these because each of these cohorts had mutation calls in at least one sample for the five genes of interest. We observed that many samples had insufficient coverage in these genes. Again as in the intercohort analysis, individual samples using a SeqCap EZ HGSC VCRome kit showed insufficient coverage in the lncRNAs. Fewer than 20% of samples in these two cohorts showed sufficient coverage of *MALAT1* and *XIST*. Therefore, these genes are particularly susceptible to underestimation of the mutation rate. Similar effects were observed for the exon capture kits Gapfiller_7m, a proprietary capture kit developed by Roche NimbleGen and used by Baylor University, and Agilent’s SureSelect Human All Exon 38 Mb v2 in these two cohorts.

### Custom V2 Exome Bait capture kit poorly covers human exome

We next sought to determine the full extent of insufficiently-covered genes associated with the Custom V2 Exome Bait and SeqCap EZ HGSC VCRome kits, as these account for 6353 and 1395 samples in TCGA respectively. We obtained the SeqCap EZ HGSC VCRome capture target BED file [12] and quantified the base-level overlap with canonical exons of TCGA genes, which we defined as genes with a called mutation in any TCGA cohort (Methods). A gene with fewer than 80% of bases overlapping between the capture target and canonical exon coordinates was initially considered undercovered. We observed 17828 undercovered genes out of the 20974 TCGA genes for which a canonical exon was identified. These included *MALAT1* and *XIST* which were due to a complete lack of coverage in their loci (S3 Fig), supporting false negative mutation calling in these genes. However, most cases of undercovered genes were due to the inclusion of untranslated regions (UTRs). When only considering the coding sequences (CDS), only 2353 genes were observed to be undercovered (Table E in S1 Tables). We also quantified the base-level probe coverage of Integrated DNA Technology’s xGen Exome Research Panel [13], a newer capture kit. We found only 873 undercovered genes (Table F in S1 Tables), likely a result of improved chemistries and synthesis technologies.

**Fig 4:**
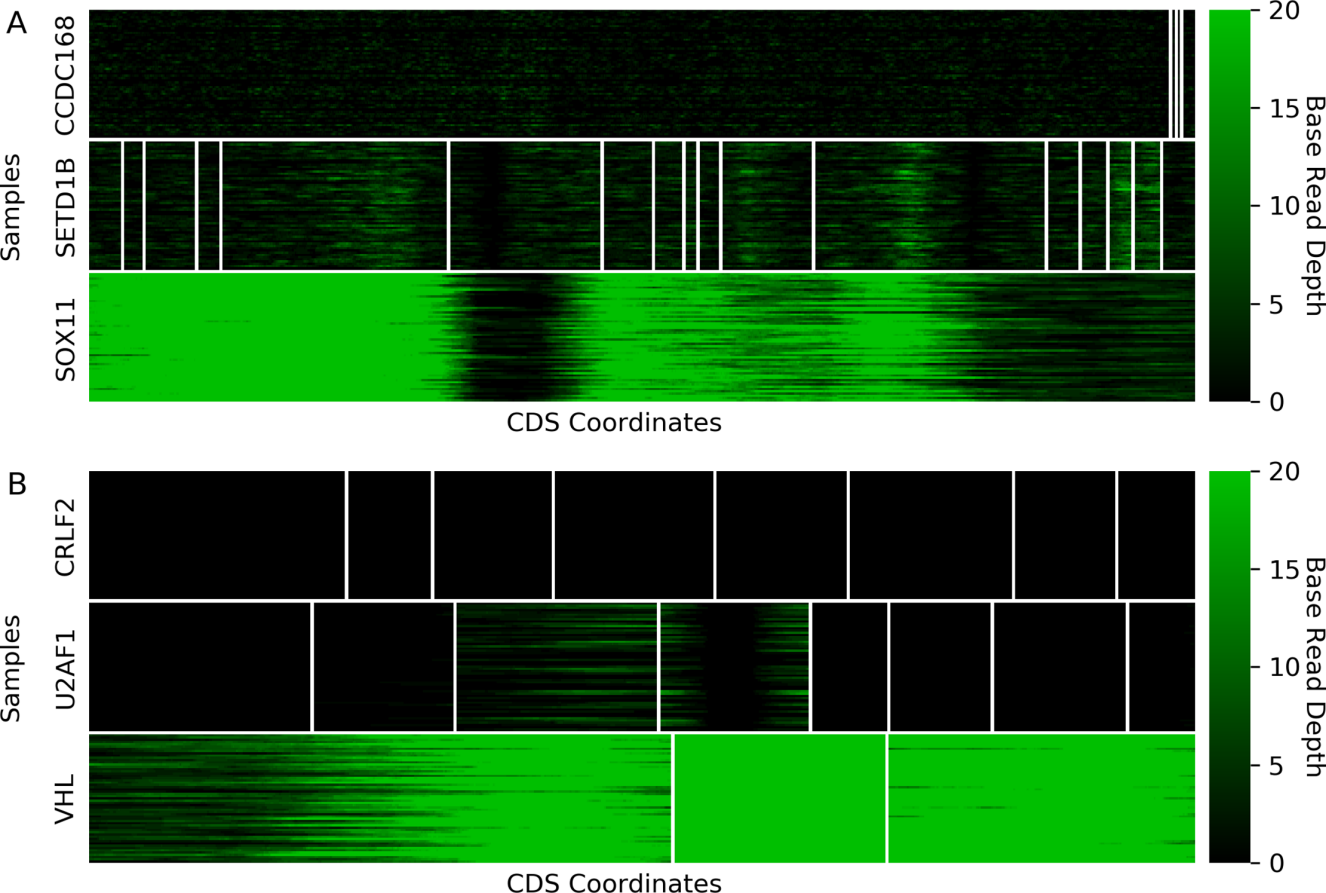
Custom V2 Exome Bait kit base coverage of select genes. Heatmaps of base-level depth in the UCS cohort for the three previously-identified undercovered genes associated with the Custom V2 Exome Bait kit (A) and three cancer genes (B). Coding exons are plotted separately to highlight absence of probe coverage, such as in *CCDC168*, or incomplete probe coverage, such as in *VHL*, across different regions. Base coordinates follow chromosomal coordinates and are limited to CDS regions.

Unlike the two previous exome capture kits, the Custom V2 Exome Bait kit does not have a publicly-available probe target design file. To find undercovered genes, we retrieved the base-level sequencing depth for TCGA genes’ canonical CDS from all samples in the Uterine Carcinosarcoma (UCS) cohort (Methods). We adjusted the definition of an undercovered gene such that 80% of bases must have an average sequencing depth greater than 20 across the 57 samples. We found 4833 undercovered genes using this modified definition, which included *CCDC168*, *SETD1B*, and *SOX11* (Table G in S1 Tables), accounting for 23% of TCGA genes. For these 4833 genes, we further applied the undercovered definition to individual exons, with an undercovered exon defined as one with fewer than 80% of bases having a sequencing depth greater than 20 (Table G in S1 Tables). *CCDC168* and *SETD1B* had uniformly low depth across all bases and exons, with no bases having an average depth greater than 20 (Fig 4A). *SOX11* had low depth over large portions of its single coding exon (Fig 4A), with 55% of bases having an average depth less than 20. These examples represent two modes for undercovered genes—either absent or incomplete capture probe coverage. Additionally, we found 1258 genes where all coding exons were undercovered.

To assess the consequences of poor capture of nearly a quarter of the human exome, we compared undercovered genes by kit to 369 known cancer genes [14]. For the Custom kit, 53 cancers genes were undercovered (Table H in S1 Tables). Only two, *CRLF2* and *U2AF1*, were a result of absent probe coverage whereas the rest, such as *VHL*, were due to incomplete probe coverage (Fig 4B). The SeqCap EZ HGSC VCRome and xGen Exome Research Panel kits had 29 and 5 undercovered cancer genes respectively with only one each with absent probe coverage (Table H in S1 Tables), in line with fewer overall undercovered genes from these two kits. While the small number of cancer genes with absent coverage is reassuring, the number of incompletely-covered cancer genes by the Custom V2 Exome Bait kit accounts for a non-trivial number of known cancer genes.

To better understand the limitations of the Custom V2 Exome Bait kit, we assessed the read depth at previously identified MSI loci [6] in the STAD cohort, another cohort which exclusively used the Custom V2 Exome Bait kit (Table C in S1 Tables). Undercoverage of several genes (*ACVR2A*, *ASTE1*, *KIAA2018*, *SLC22A9*, and *TGFBR2)* were common events across multiple tumor types, including STAD. In each of these cases, such as with *ACVR2A* (A in S4 Fig), the average read depth within the MSI loci across all TCGA-STAD samples was greater than 60 (Table I in S1 Tables), indicating that these are likely true-positive MSI loci. *CCDC168*, *SMAP1*, and *SPINK5* were less common MSI events not seen in STAD. As previously shown, the coverage at the *CCDC168* locus was poor and explained the lack of MSI events. The two *SMAP1* MSI loci had an average read depth of 27.3 and 11.2 (Table I in S1 Tables), much lower in comparison to the rest of the genetic loci (B in S4 Fig) and suggesting another false-negative MSI location in STAD. Coverage at and around MSI loci is important as alignment discrepancies due to slippage could lead to spurious single nucleotide variant calls. We note that current TCGA pipelines do ameliorate this effect (S5 Fig). The three *SPINK5* MSI loci had average read depths of 97.5, 179.2, and 20.9 (Table I in S1 Tables and C in S4 Fig). MSI loci previously identified as absent in STAD may be false negatives as a result of poor read coverage.

## Discussion

We have demonstrated that choice of exon capture kit systematically impacts mutation calling in a cohort-dependent manner, and in particular we considered five genes as case studies that were repeatedly uncalled across diverse cohorts even at stringent filtering criteria. These effects are due to insufficient coverage associated with poor capture. Although we expected some variability in exome capture efficiency between methods [15] and heterogeneous gene coverage across samples [16], our study reveals strong biases in TCGA that have not been previously reported. 6353 samples, i.e. over half of TCGA, were assayed with the Custom V2 Exome Bait kit which we found to undercover at least 4833 genes. As only a few mutations drive any given tumor [14], these methodological issues have the potential to substantially alter the understanding of a patient’s tumor genetics.

All of the 5 undercovered genes that we initially identified have known or presumed roles in cancer. For example, overexpression of *MALAT1*, i.e. metastasis-associated lung adenocarcinoma transcript 1, is known to be associated with metastasis markers in non-small cell lung cancer [17,18] and colorectal cancer [19]. Reducing *MALAT1* expression leads to reduced growth and metastasis in bladder cancer mouse models [20], making it a potential therapeutic target. *SOX11 is* a tumor suppressor in glioma [21] and prognostic marker in epithelial ovarian cancer [22] among other roles. Its primary mechanism in tumorigenesis is silencing by DNA methylation [23]. Such an alteration is actionable though, as epigenetic modifiers such as the DNA methyltransferase inhibitor 5-Aza-dC have been shown to increase expression of *SOX11* and slow growth [24].

*XIST* has been shown to have a role in several cancer types. Deletion of *XIST* in blood cells of female mice results in X reactivation and blood neoplasms [25]. *XIST* has also been proposed to act as a tumor suppressor in breast cancer by regulating phosphorylated AKT [26], and as an oncogene in non-small cell lung cancer by downregulating the tumor suppressor KLF2 [27]. *SETD1B* mutations have been found in multiple cancers [28]. Frameshift mutations commonly occur at the locus, likely related to microsatellite instability [29] in ways similar to *CCDC168*. *SETD1B* is part of the H3K4 methyltransferase family KMT2, in which fusion events in several members are implicated as drivers in mixed lineage leukemia (MLL) [30]. Histone modification in colon cancer also relates to tumorigenic transcriptional signatures [31], although not necessarily causally as in MLL.

The finding of poor coverage at 4833 genes, including 53 known cancer genes, by the Custom V2 Exome Bait kit presents an important problem to be aware of in cancer genomic analysis. 1258 of these genes have absent or poor probe coverage spanning the entire coding region, a nontrivial fraction of the genome with little interpretable information. Kit dependencies can bias comparisons between tumor histologies, and likely explain a prior report that *CCDC168* and *SMAP1* are sites of microsatellite instability in colon adenocarcinoma but not stomach adenocarcinoma [6]. Low coverage could exacerbate variant mis-calling, particularly if poorly-covered regions are subject to alignment issues as might be expected at MSI loci. However, prior studies have shown that local realignment near indels has improved the ability filter out false-positive SNVs in these regions [32]. The remaining 3575 genes with poor coverage of specific coding regions also constitute a sizeable fraction of the exome. For these, specific mutations within a gene may be underreported, and driver mutation differences between cancer types may be inappropriately identified. In both situations, technical sequencing artifacts are strong confounders preventing true interpretation of genetic differences between tumor histologies.

Several strategies should be considered to allow for comparison between samples and cohorts assayed with separate capture kits. The first would be to restrict analyses to regions and genes where sufficient reads occur in both groups, reducing the occurrence of falsely-identified differences. This compromise is acceptable for experiments using newer exome kits where the union of poorly-covered regions will be smaller but may limit analyses with TCGA cohorts using the Custom V2 Exome Bait kit. Another potential strategy would be to pool reads in poorly-covered regions at the cohort level to rescue mutation calls. This would allow for some comparisons that include Custom V2 Exome Bait kit cohorts without discarding information at the expense of cohort-level resolution as opposed to sample-level resolution. The drawback for this method is the increased rate of false-positive associations in order to increase the true-positive rate.

In summary, our findings reveal strong undercalling of TCGA mutations in cancer genes due to problems in capturing their exons for sequencing. The five genes that we focused on are merely the most extreme of more systematic biases, as we found at least 4833 other genes that are undercalled in the samples assayed by the Custom V2 Exome Bait capture kit and 2353 in samples assayed by the SeqCap EZ HGSC VCRome capture kit. Such biases may hide shared driver mechanisms in tumors of different histologies, obscuring key differences and similarities between tumors as well as samples within cohorts. In both cases, this would lead to spurious subtyping based on mutational status. TCGA is an invaluable resource for understanding the genetics of cancer, but it is important to be cognizant of its biases. Otherwise measurement issues such as choice of exome capture kit will confound attempts at broad understanding across cancers.

## Methods

### Filtering initial genes of interest

To identify genes with potential false negative mutation calls, we analyzed each TCGA cohort’s MAF file obtained from the Genomic Data Commons (GDC). We searched for genes with at least a minimal mutation rate across all cohorts and then identified cohorts that appeared to have a spurious lack of called mutations for that gene. For each non-silent mutation, we looked for cohorts where zero patients have a called mutation in a gene. As a first filtering step, we eliminated rarely mutated genes by retaining only those with at least a 5% mutation rate across cohorts having non-zero mutation calls. There are also many small cohorts where it would not be unlikely for a gene to have zero called mutations, even at a 5% true mutation rate. Therefore only genes where three or more cohorts had no called mutations were considered further, eliminating cases isolated to smaller cohorts. The remaining 136 genes are shown with their respective sets of cohorts lacking mutation calls in Table B in S1 Tables.

### Filtering genes for interrogating Custom V2 capture kit

To identify all genes affected by the Custom V2 Exome Bait capture kit, we chose genes with no called mutations in the 14 cohorts using the kit exclusively. Only those genes which had an associated Gencode v22 name were chosen to probe further as GDC’s BAM Slicing API uses Gencode v22 gene names to retrieve reads.

### Retrieving gene-specific reads

We used GDC’s BAM Slicing API to download gene-specific reads for each sample based on the gene-cohort associations in Table B in S1 Tables, for all cohorts for the five genes of interest, and for the seven additional MSI loci. For retrieving reads from all TCGA genes, we instead downloaded all whole-exome BAM files for the TCGA-UCS cohort from GDC’s Data Portal. We processed reads using samtools version 1.5 [33] to discard duplicated reads and those below a mapping quality of 30, calculate the average exon coverage using coordinates of UCSC canonical exons (S1 Data) and determine base-level depth across all TCGA genes. Canonical exons were retrieved from UCSC Genome Browser’s Table Browser using assembly GRCh38, track GENCODE v24, group Genes and Gene Predictions, and table knownCanonical. Average exon coverage results for cohorts can be found in S3.

### Querying TCGA sample information

We used GDC’s Search and Retrieval API to query TCGA sample information for all cohorts. We restricted our query to the WES BAM files for tumor samples only. Retrieved information consisted of filename hash ids necessary for the BAM Slicing API and metadata regarding the whole-exome capture kit used for sequencing.

### Capture target BED comparison versus canonical exons

BED files obtained from the respective manufacturer’s websites were converted from the hg19 genome assembly to hg38 with UCSC Genome Browser’s LiftOver, using the default webtool parameters. TCGA genes, defined as a gene with a called mutation in any TCGA cohort, were identified by aggregating all mutations across all 33 tumor types’ MAF files. Exon and CDS coordinates were drawn from canonical exons used previously and retrieved from UCSC Genome Browser’s Table Browser using assembly GRCh38, track GENCODE v24, group Genes and Gene Predictions, and table knownGenes. Of the 22042 genes with a called mutation, 20974 mapped to UCSC canonical exons.

### Code and data availability

Bash and Python scripts to perform the work in this manuscript are available online at https://github.com/TheJacksonLaboratory/GDCSlicing. Bed files of canonical exon coordinates are available as a supplemental file (S1 Data). Calculated average exon coverage data for TCGA samples corresponding to Figs 2 and 3 is available as a supplementary file (S2 Data). MSI loci depth data corresponding to Table H in S1 Tables are available as a supplemental file (S3 Data). Calculated base-level depth data for TCGA-UCS samples corresponding to Fig 4 is available upon request.

## Supporting information

S1 Fig Sparse sequencing coverage on *CCDC168*. IGV plot of reads aligned to the *CCDC168* locus for sample TCGA-D7-6518 in STAD. After filtering, only 12 reads align to the gene, and this is the median number for samples in STAD. For reference, microsatellite instability (MSI) loci previously identified in TCGA [6] are also shown in the second track. The few reads aligning to *CCDC168* in this sample do not overlap well with the MSI loci.
(TIF)

S2 Fig Whole cohorts with insufficient coverage. The STAD cohort (A) had no samples with sufficient coverage of the *CCDC168* locus. Other genes share this same behavior, namely *SETD1B*, and *SOX11* in the PRAD cohort (B), and *MALAT1* and *XIST* in the LIHC cohort (C).
(TIF)

S3 Fig Undercovered genes likely due to exome capture protocol design. The VCRome exome capture kit does not contain probes for the loci containing *MALAT1* (A) and *XIST* (B), corresponding to the poor depth in samples using the kit. On the contrary, the VCRome kit does contain probes for *CCDC168* (C) which does have reads in samples using this kit.
(TIF)

S4 Fig ACVR2A (A), a previously-identified stomach adenocarcinoma MSI loci, appears to have reasonable coverage of the MSI region (third track) in two TCGA-STAD samples. SMAP1 (B) and SPINK5 (C) are MSI loci associated with colorectal adenocarcinoma but not stomach adenocarcinoma. TCGA-STAD samples appear to have poor coverage of the SMAP1 MSI loci whereas the SPINK5 appears to have much higher coverage of the MSI loci.
(TIF)

S5 Fig TCGA standard alignment approaches correctly handle microsatellite instability loci for SNV/indel calling. The co-cleaning step in the Genomic Data Commons pipeline that incorporates local realignment around indels handles this issue. For example, we performed BWA alignment of reads to microsatellite instability loci in CCDC168 for TCGA-COAD sample TCGA-AD-6889 without the co-cleaning step (top read track). Reads are grouped in 3 sets based on their nucleotide at a known indel (vertical purple bar). The red circle indicates a locus with multiple read support for an SNV prior to co-cleaning. After co-cleaning (bottom read track), these reads no longer support SNV status.
(TIF)

S1 Tables Supplemental Tables A-I Table A. STAD CCDC168 Coverage
Table B. Genes of Interest
Table C. Capture Kit by Cohort
Table D. Capture Kit Frequency by Cohort
Table E. SeqCap EZ HGSC VCRome Undercovered Genes and Exons
Table F. xGen Exome Research Panel Undercovered Genes and Exons
Table G. Custom V2 Exome Bait Undercovered Genes and Exons
Table H. Undercovered Cancer Genes and Exons
Table I. STAD MSI Loci.
(XLSX)

S1 Data Bed files for canonical exons of TCGA genes. (ZIP)

S2 Data Calculated exon coverage from TCGA samples used to generate figures and tables. (ZIP)

S3 Data Calculated base depth of MSI loci for TCGA-STAD samples used to generate Table H in S1 Tables. (ZIP)
