# Supplementary figures and images for: Whole-exome sequencing capture kit biases yield false negative mutation calls in TCGA cohorts
Source: PLoS One. 2018 Oct 3;13(10):e0204912. doi: 10.1371/journal.pone.0204912 (PMC6169918; doi:10.1371/journal.pone.0204912)

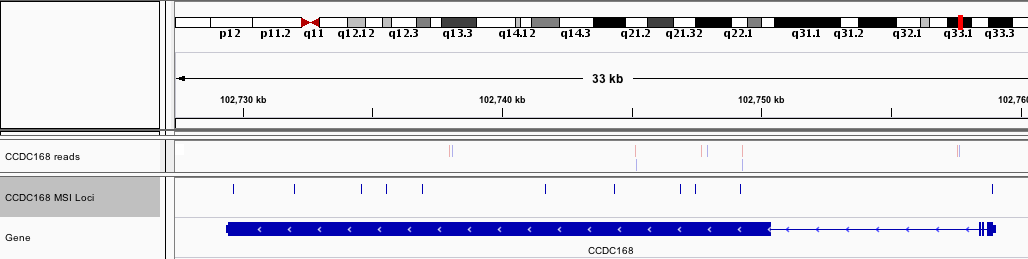

Supplement: S1 Fig — IGV plot of reads aligned to the CCDC168 locus for sample TCGA-D7-6518 in STAD. After filtering, only 12 reads align to the gene, and this is the median number for samples in STAD. For reference, microsatellite instability (MSI) loci previously identified in TCGA [6] are also shown in the second track. The few reads aligning to CCDC168 in this sample do not overlap well with the MSI loci. (TIF) [file pone.0204912.s001.tif]

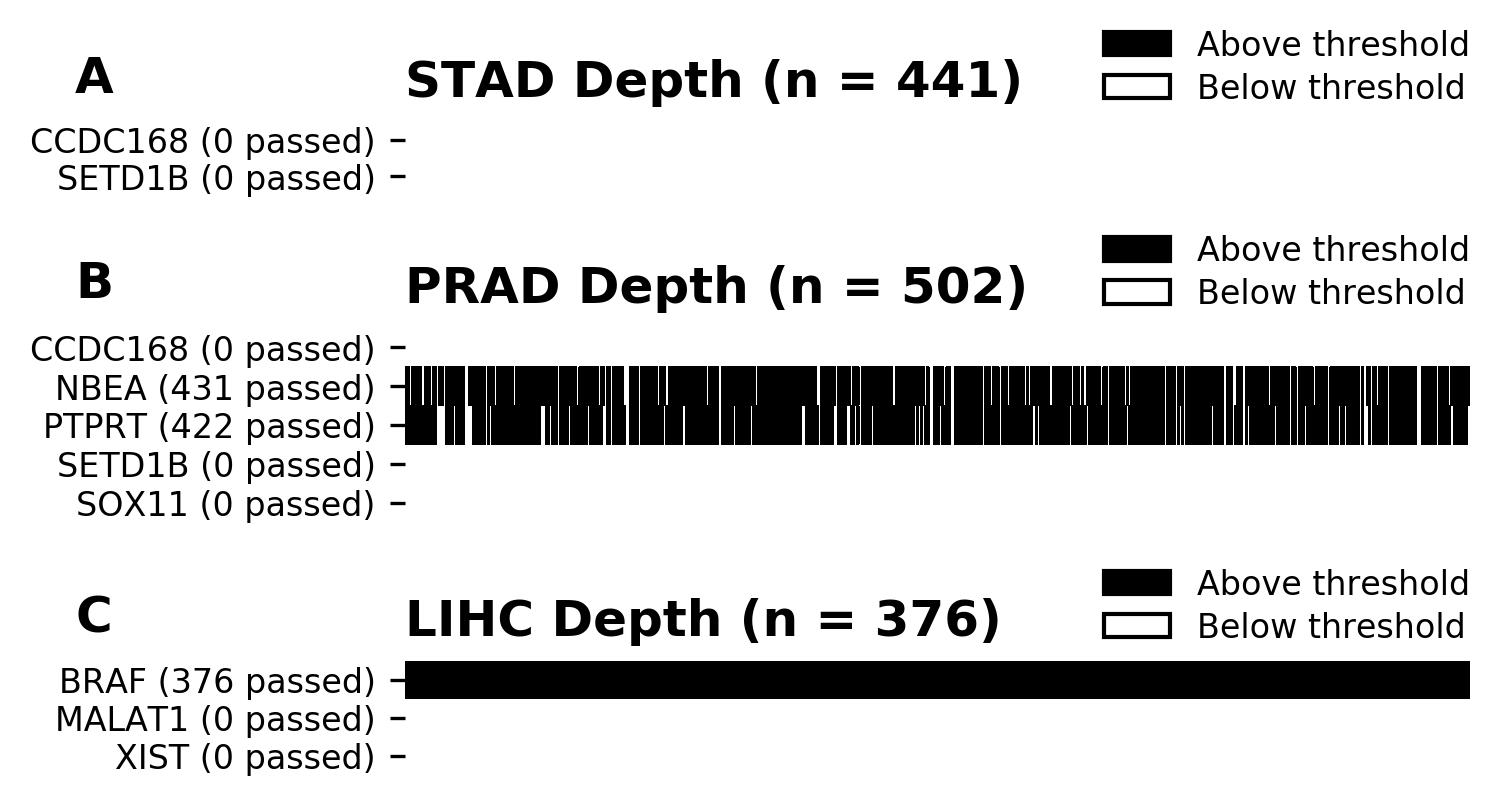

Supplement: S2 Fig — The STAD cohort (A) had no samples with sufficient coverage of the CCDC168 locus. Other genes share this same behavior, namely SETD1B, and SOX11 in the PRAD cohort (B), and MALAT1 and XIST in the LIHC cohort (C). (TIF) [file pone.0204912.s002.tif]

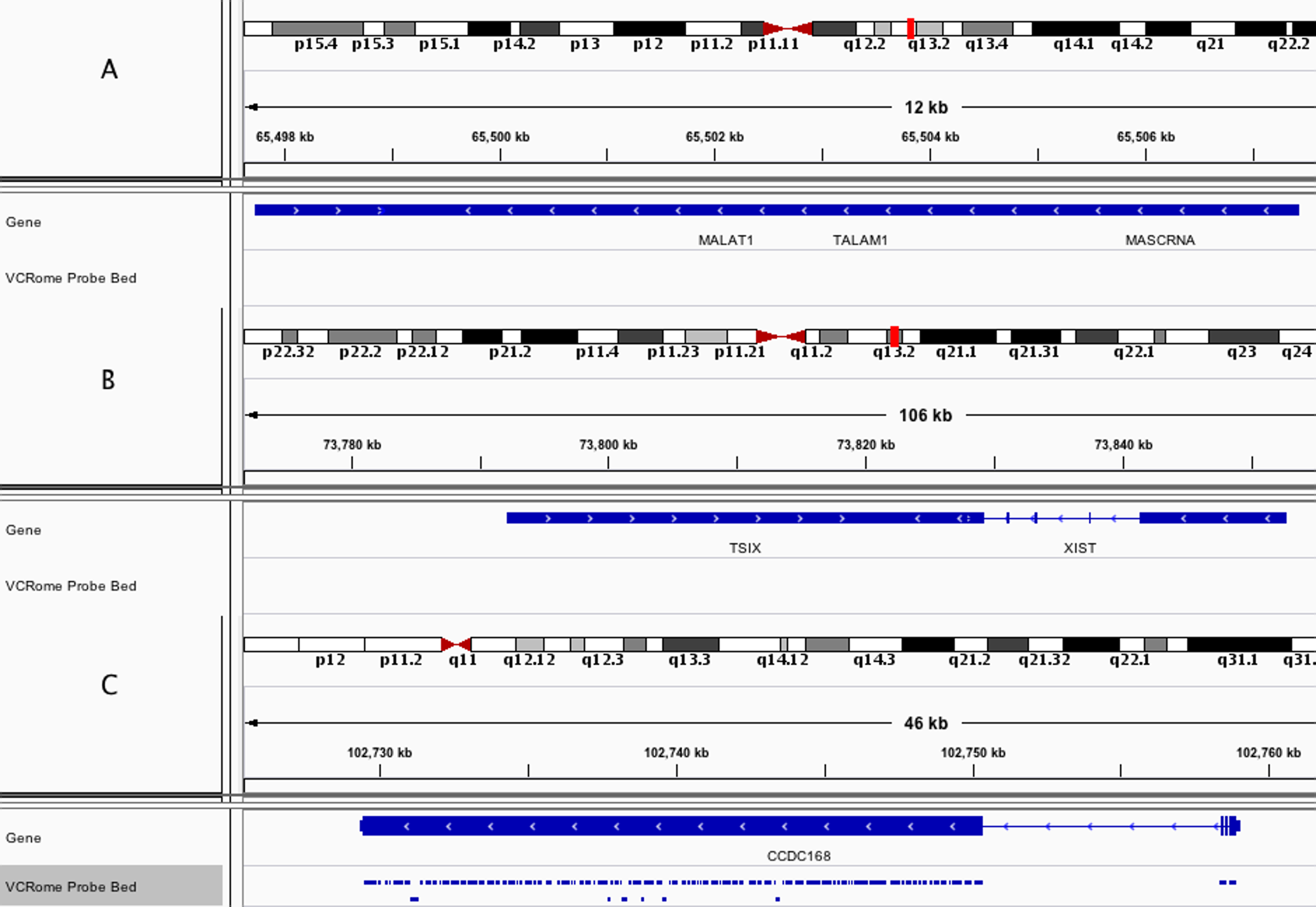

Supplement: S3 Fig — The VCRome exome capture kit does not contain probes for the loci containing MALAT1 (A) and XIST (B), corresponding to the poor depth in samples using the kit. On the contrary, the VCRome kit does contain probes for CCDC168 (C) which does have reads in samples using this kit. (TIF) [file pone.0204912.s003.tif]

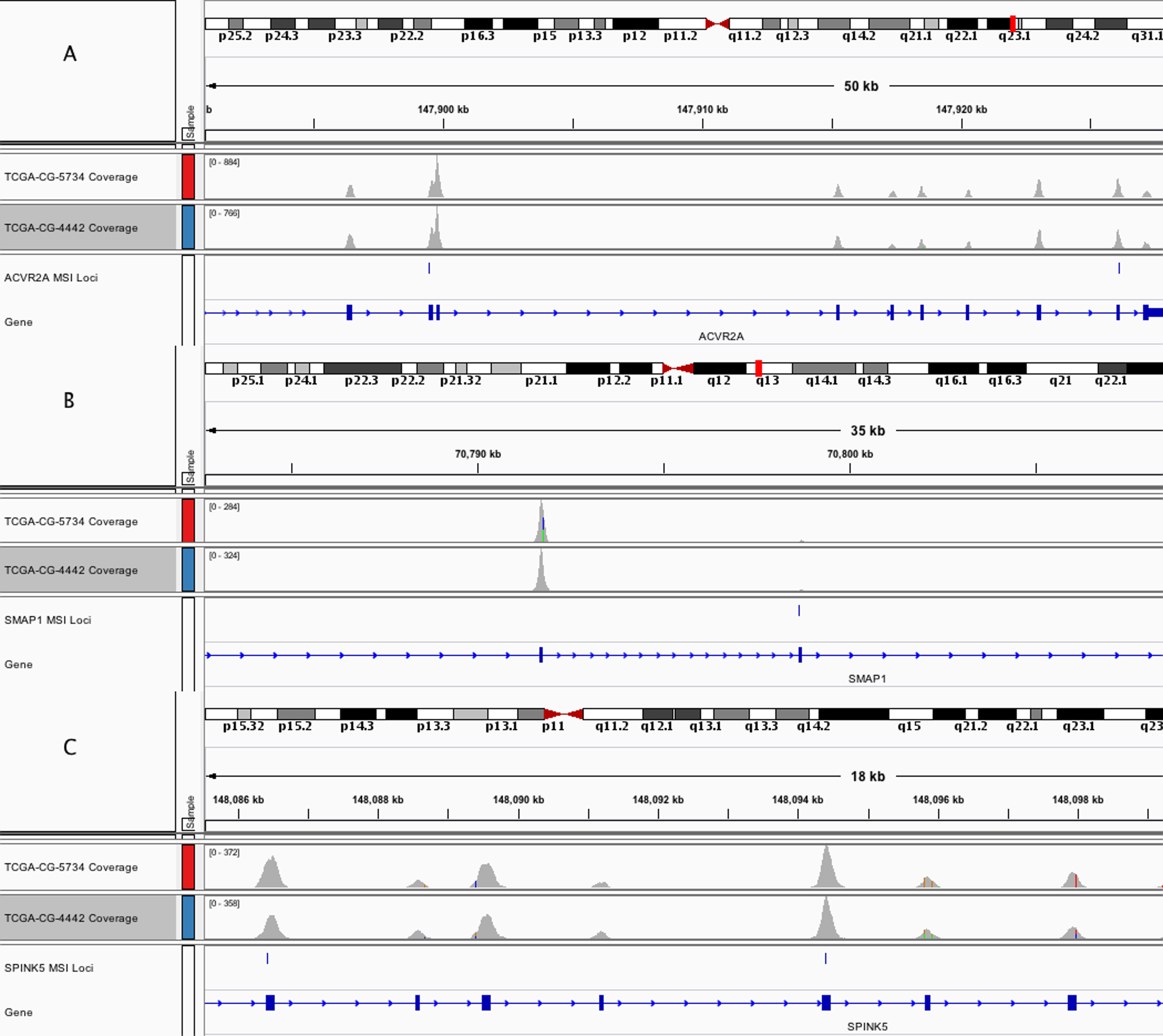

Supplement: S4 Fig — ACVR2A (A), a previously-identified stomach adenocarcinoma MSI loci, appears to have reasonable coverage of the MSI region (third track) in two TCGA-STAD samples. SMAP1 (B) and SPINK5 (C) are MSI loci associated with colorectal adenocarcinoma but not stomach adenocarcinoma. TCGA-STAD samples appear to have poor coverage of the SMAP1 MSI loci whereas the SPINK5 appears to have much higher coverage of the MSI loci. (TIF) [file pone.0204912.s004.tif]

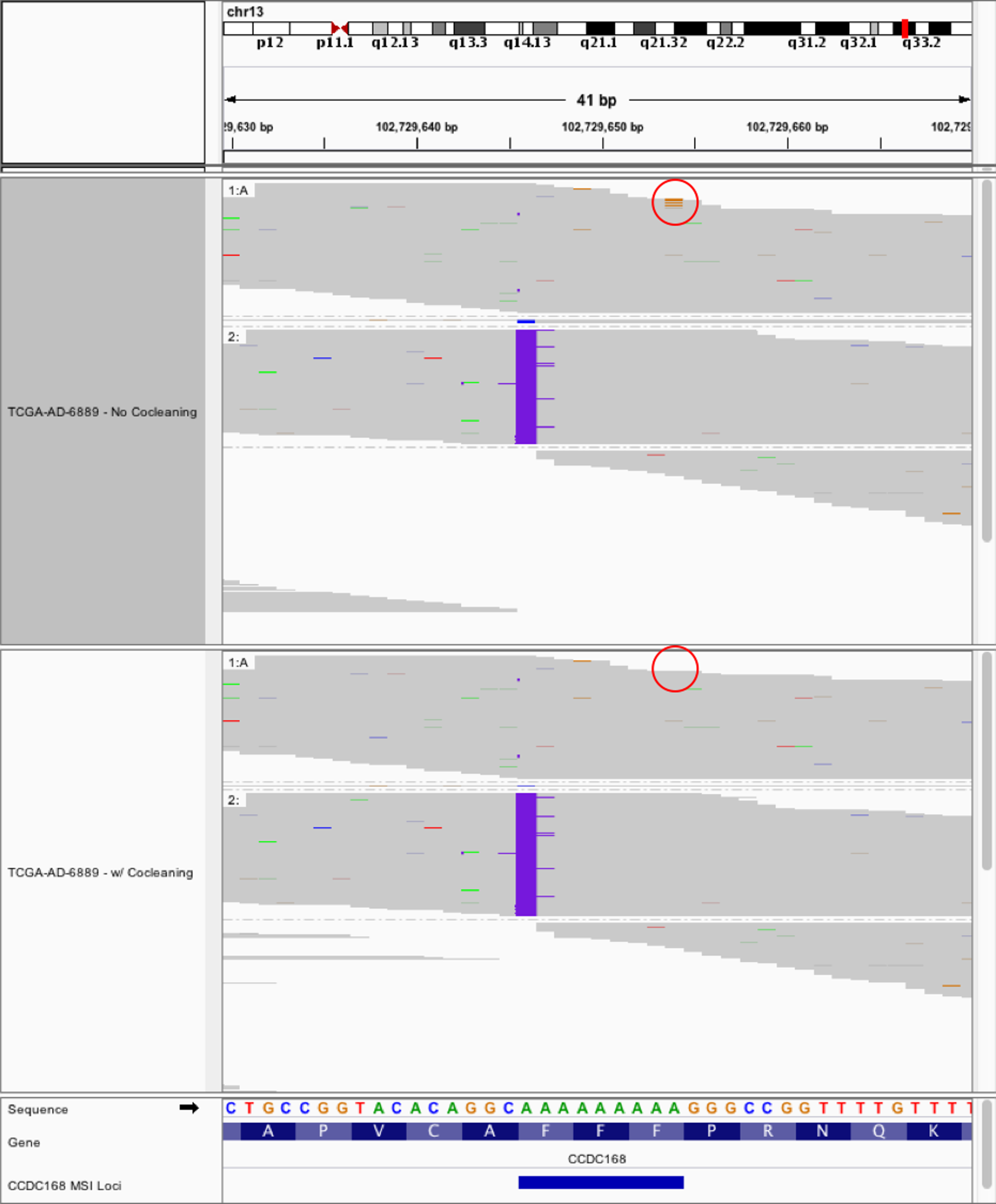

Supplement: S5 Fig — The co-cleaning step in the Genomic Data Commons pipeline that incorporates local realignment around indels handles this issue. For example, we performed BWA alignment of reads to microsatellite instability loci in CCDC168 for TCGA-COAD sample TCGA-AD-6889 without the co-cleaning step (top read track). Reads are grouped in 3 sets based on their nucleotide at a known indel (vertical purple bar). The red circle indicates a locus with multiple read support for an SNV prior to co-cleaning. After co-cleaning (bottom read track), these reads no longer support SNV status. (TIF) [file pone.0204912.s005.tif]
